# A Critical Role for FBXW8 and MAPK in Cyclin D1 Degradation and Cancer Cell Proliferation

**DOI:** 10.1371/journal.pone.0000128

**Published:** 2006-12-27

**Authors:** Hiroshi Okabe, Sang-Hyun Lee, Janyaporn Phuchareon, Donna G. Albertson, Frank McCormick, Osamu Tetsu

**Affiliations:** 1 Department of Pathology, School of Medicine, University of California San Francisco, San Francisco, California, United States of America; 2 Cancer Research Institute, School of Medicine, University of California San Francisco, San Francisco, California, United States of America; 3 UCSF Comprehensive Cancer Center, School of Medicine, University of California San Francisco, San Francisco, California, United States of America; Department of Biochemistry, China

## Abstract

Cyclin D1 regulates G1 progression. Its transcriptional regulation is well understood. However, the mechanism underlying cyclin D1 ubiquitination and its subsequent degradation is not yet clear. We report that cyclin D1 undergoes increased degradation in the cytoplasm during S phase in a variety of cancer cells. This is mediated by phosphorylation at Thr286 through the activity of the Ras/Raf/MEK/ERK cascade and the F-box protein FBXW8, which is an E3 ligase. The majority of FBXW8 is expressed in the cytoplasm during G1 and S phase. In contrast, cyclin D1 accumulates in the nucleus during G1 phase and exits into the cytoplasm in S phase. Increased cyclin D1 degradation is linked to association with FBXW8 in the cytoplasm, and enhanced phosphorylation of cyclin D1 through sustained ERK1/2 signaling. Depletion of FBXW8 caused a significant accumulation of cyclin D1, as well as sequestration of CDK1 in the cytoplasm. This resulted in a severe reduction of cell proliferation. These effects could be rescued by constitutive nuclear expression of cyclin D1-T286A. Thus, FBXW8 plays an essential role in cancer cell proliferation through proteolysis of cyclin D1. It may present new opportunities to develop therapies targeting destruction of cyclin D1 or its regulator E3 ligase selectively.

## Introduction

Cyclin D1 regulates G1 progression in cancer cells and is overexpressed in various malignant neoplasms [1]. As a result, it is a potential target for cancer therapeutics [2]. Transcriptional regulation of cyclin D1 has been extensively studied and is well understood [3]–[5]. It is stimulated when, for example, various mitogenic signals activate the Ras/Raf/MEK/ERK (MAPK) cascade. After synthesis following MAPK cascade activation, cyclin D1 associates with CDK4/6, p21 Cip1 or p27 Kip1 [6], [7].

In contrast, the mechanism of cyclin D1 ubiquitination and subsequent degradation has not been fully characterized. It is known that cyclin D1 is polyubiquitinated and subsequently degraded through the 26S proteasome pathway. This process requires phosphorylating cyclin D1 at threonine (Thr)-286, which is located near its C terminus [8]. The cyclin D1 mutant T286A is resistant to ubiquitination *in vitro* and *in vivo* and is a highly stable protein. Glycogen synthase kinase-3β (GSK3β) can phosphorylate cyclin D1 at Thr286, promoting nuclear-to-cytoplasmic redistribution of cyclin D1 [9], [10]. However, the role of GSK3β in cyclin D1 phosphorylation and its stability have been questioned recently [11], [12], and p38 SAPK2 has been implicated in proteasomal degradation of cyclin D1 following osmotic shock [13].

We attempted to identify the kinase responsible for phosphorylating cyclin D1 at Thr286. Our work shows that cyclin D1 is destabilized specifically during S phase in cancer cells and that increased degradation is mediated by phosphorylation at Thr286 through the activity of the Ras/Raf/MEK/MAPK signaling cascade.

The ubiquitin-protein ligase necessary for degrading cyclin D1 has not yet been identified. An F-box protein, SKP2, has been proposed [14], [15]. However, SKP2-mediated regulation of cyclin D1 may be context- or cell line dependent, and may be indirect [7]. Furthermore, cyclin D1 does not accumulate in SKP2 knockout mouse embryonic fibroblast (MEF) cells [16], [17].

The formation of polyubiquitin-protein conjugates is well-understood [18]. Three components participate in sequential ubiquitin transfer reactions: E1, an activating enzyme, E2/Ubc, a ubiquitin-conjugating enzyme, and E3, a protein ligase, which attaches ubiquitin to a lysine residue on a target protein.

The best characterized of these enzymes are the SCF E3 ubiquitin ligases, which regulate substrate ubiquitination in a phosphorylation-dependent manner [19]–[21]. These ligases form a highly diverse family of complexes named for their components, S-phase Kinase-associated Protein 1 (SKP1), Cullin 1 (CUL1/Cdc53), F-box proteins, and RBX1/ROC1. SKP1 is a crucial adaptor subunit and selectively interacts with a scaffold protein, either CUL1 or Cullin 7 (CUL7), to promote the ubiquitination of targeted substrates [22], [23]. Association of CUL7 with SKP1 depends on FBXW8 and forms a specific SCF-like complex [22], [23]. Our study demonstrates that the F-box protein FBXW8 specifically recognizes the cyclin D1 in a phosphorylation-dependent manner and regulates its stability through the ubiquitin-proteasome pathway.

## Results

### Cyclin D1 protein is destabilized specifically in S phase in cancer cells

**Figure 1:**
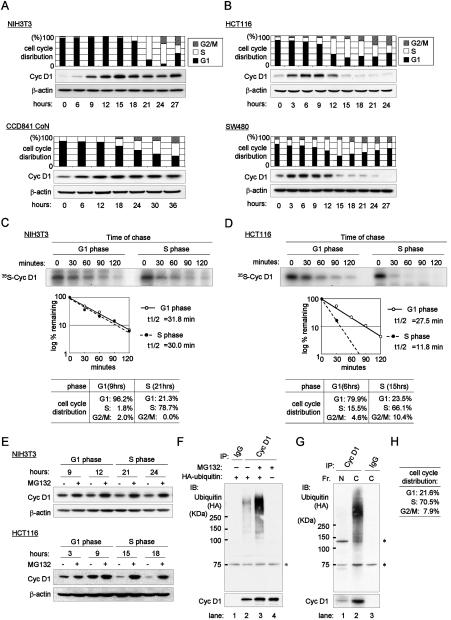
Cyclin D1 is destabilized during S phase through the ubiquitin-proteasome pathway in cancer cells. (A, B) Expression profile of cyclin D1 during cell cycle progression in cells released from quiescence. Normal cells (A) and cancer cells (B) were tested. (A) NIH 3T3 mouse fibroblasts and CCD841 CoN normal colon epithelium. (B) HCT 116 and SW480 cells. CCD841 CoN cells were synchronized at G0/G1 phase by treatment with ACL-4 media without EGF for 24 hrs, and then stimulated with complete ACL-4 media containing EGF. Other cells were released from quiescence by serum stimulation. Samples were collected at indicated time points. Cell cycle distributions were determined by flow-cytometry and percentages of each phase are indicated. Western-blots were performed with cyclin D1 and β-actin antibodies, respectively. (C, D) Pulse-chase analysis of cyclin D1 in NIH 3T3 cells (C) and HCT 116 cells (D). Cells were released from quiescence and labeled with ^35^S-methionine for 1 hour when most of the populations were in G1 phase (9 hrs for NIH3T3 and 6 hrs for HCT 116) or in S phase (21 hrs for NIH3T3 and 15 hrs for HCT 116). The cells were chased with cold methionine for the indicated times and then lysed. Cyclin D1 was immunoprecipitated and analyzed with SDS-PAGE. Autoradiography was performed. Levels of metabolically labeled-cyclin D1 were estimated by quantitative scanning using Quantity One software (Bio-Rad) and blotted on the graph to determine the half-life of cyclin D1. Cell cycle distribution is indicated below the figures. (E) Turnover of cyclin D1 is mediated by the ubiquitin-proteasome pathway. NIH3T3 and HCT 116 cells were released from quiescence by serum stimulation and treated in the presence (+) or absence (−) of MG132 for 2 hrs prior to harvesting at each time point. Western blot was performed with cyclin D1 and β-actin antibodies. (F–H) Polyubiquitination of cyclin D1. (F) HCT 116 colon cancer cells were transfected with HA-tagged ubiquitin cDNA (lanes 1–3) or without it (lane 4) and then synchronized to S phase through the sequential manipulation of serum starvation and stimulation. Cells were treated with 25 µM MG132 (lanes 3 and 4) or without it (lanes 1 and 2) for one hour. Lysates were immunoprecipitated with antibodies to cyclin D1 antibody (lanes 2–4) or control IgG (lane 1) and immunoblotted with a HA antibody (upper panel) or a cyclin D1 antibody (lower panel). Asterisks indicate background non-specific bands (F–G). (G) Nuclear (N) and cytoplasmic (C) proteins were fractionated (Fr.) from cell lysates collected in Panel F, lane 3. Nuclear and cytoplasmic extracts were immunoprecipitated with antibodies to cyclin D1 (lanes 1 and 2) or IgG (lane 3) and immunoblotted with a HA antibody (upper panel) or a cyclin D1 antibody (lower panel). (H) Cell cycle distributions.

To investigate the mechanism and importance of cyclin D1 proteolysis, we first assessed the expression profile of cyclin D1 during cell cycle progression from quiescence in three normal cell lines (NIH 3T3 & WI-38 fibroblasts, and CCD841 CoN colon epithelium cells) and in three cancer cell lines (HCT 116 and SW480 colon cancers and T98G glioblastomas). Normal cells (Fig. 1A and Fig. S1 [A]) and cancer cells (Fig. 1B and Fig. S1 [B]) were released from quiescence at G0/G1 phase and cell cycle profiles were determined by flow-cytometric cell cycle analyses. In both cell types, cyclin D1 expression gradually increased after re-entry into the cell cycle and reached a maximum at the G1-S transition. In all three normal cell lines, cyclin D1 levels remained constant during S phase (Fig. 1A and Fig. S1 [A]), although we observed a slight decrease in cyclin D1 expression after entry into S phase. This finding is consistent with previous observations [24], [25]. In contrast, all three cancer cell lines showed a dramatic reduction of cyclin D1 expression during S phase (Fig. 1B and Fig. S1 [B]). These observations suggest that cyclin D1 turnover is increased during S phase in these cells.

To confirm this observation, NIH 3T3 mouse fibroblast and HCT 116 colon cancer cells were synchronized at the G0/G1 phase and released from quiescence and subjected to pulse-chase analysis. Figs. 1C and D show pulse-chase analyses on ^35^S metabolically labeled cyclin D1 at 9 hrs (NIH 3T3) or 6 hrs (HCT 116), when most of the cells were in G1 phase, and at 21 hrs (NIH 3T3) and 15 hrs (HCT 116) when most were in S phase (Figs. 1C and D, bottom tables). Labeled cyclin D1 levels of were estimated by quantitative scanning (Figs. 1C and D). There was no significant difference in the half-life of cyclin D1 during G1 and S phases in the NIH 3T3 cells. In contrast, there was a significant difference in the HCT 116 cells (S phase T_1/2_ =  11.8 min, compared to T_1/2_ = 27.5 min in G1 phase). Thus, cyclin D1 is destabilized specifically in S phase in HCT 116 cells.

### Cyclin D1 is degraded in the cytoplasm during S phase through the ubiquitin-proteasome pathway in cancer cells

We next determined if cyclin D1 destabilization during S phase was due to increased proteolysis through the ubiquitin-proteasome pathway. We treated HCT 116 and NIH 3T3 cells with MG132, a proteasome inhibitor, for 2 hrs prior to harvesting at each time point during cell cycle progression (Fig. 1E). In treated HCT 116 cells, cyclin D1 accumulated significantly in S phase, with no significant accumulation during G1 phase. In contrast, the difference in accumulated cyclin D1 between G1 and S phase in NIH 3T3 cells appeared to be much smaller. These findings suggest that, in these cancer cells, cyclin D1 is destabilized during S phase through the 26S proteasome pathway.

Figures 1F–H confirm that destabilization of cyclin D1 involves polyubiquitination. In Fig. 1F, HCT 116 cells were transfected with (lanes 1–3) or without (lane 4) HA-tagged ubiquitin cDNA and then synchronized to S phase. Cells were treated with MG132 (lanes 3 and 4) or without it (lanes 1 and 2) for an hour. Lysates were immunoprecipitated with a cyclin D1 antibody (lanes 2–4) or control IgG (lane 1) and immunoblotted with a HA antibody. A group of slower migrating bands was detected by the HA antibody exclusively in the anti-cyclin D1 immunoprecipitates in the presence of ubiquitin (lanes 2 and 3) and the reduced mobility bands were enhanced further after exposure to MG132 (lane 3), indicating that these bands included polyubiquitinated cyclin D1. These observations confirmed that cyclin D1 is degraded during S phase through the ubiquitin-proteasome pathway in HCT 116 cells. Fig. 1H shows that more than 70% of these cells were in S phase.

To identify where cyclin D1 degradation is increased during S phase, we extracted nuclear (N) and cytoplasmic (C) protein from cell lysates collected in Fig. 1F lane 3 (Fig. 1G). Histone H1 was exclusively detected in the nuclear fraction, whereas MEK1 was totally expressed in the cytoplasmic extract, suggesting that we successfully fractionated cell lysates (Fig. S1 [C]). The majority of cyclin D1 was localized in the cytoplasm (Fig. S1 [C]). Nuclear and cytoplasmic extracts were immunoprecipitated with antibodies to cyclin D1 (Fig 1G, lanes 1 and 2) or IgG (lane 3) and immunoblotted with a HA antibody. Polyubiquitinated cyclin D1 bands were predominantly detected in the cytoplasmic extracts. Furthermore, inhibition of nuclear-to-cytoplasmic localization of cyclin D1 with Leptomycin B (LMB) did not enhance these bands significantly in the nucleus (Fig. S1 [D]). We conclude that cyclin D1 is degraded in the cytoplasm specifically in S phase by a proteasome-dependent mechanism in HCT 116 cells.

### MAPK interacts with cyclin D1 through a D-domain and phosphorylates Thr286

**Figure 2:**
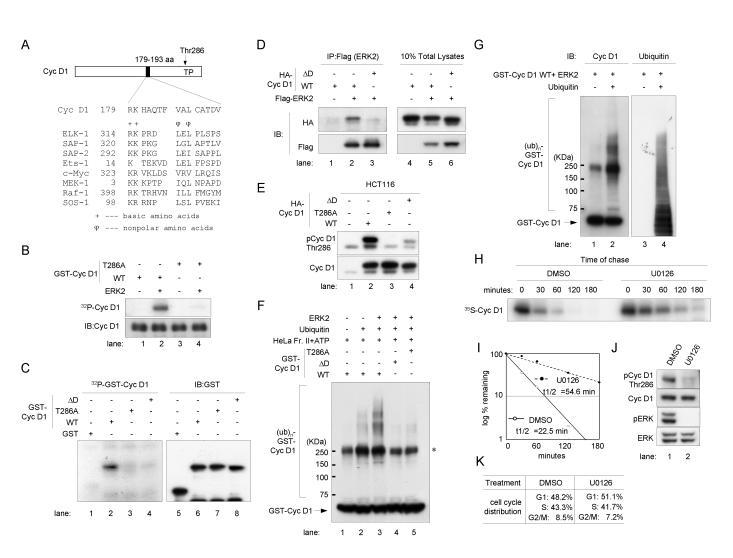
MAPK phosphorylates cyclin D1 at Thr286, which triggers subsequent ubiquitination. (A) Identification of the D-domain in cyclin D1 (Cyc D1). The illustration of full-length human Cyc D1 shows the region of the D-domain (A.A. 179-193) and the MAPK phosphorylation site Thr (T) 286 followed by proline (P) (solid bar and an arrow, respectively). The amino acid sequence of the D-domain within Cyc D1 is aligned with other known MAPK-docking sites of various ERK substrates. The doublet of basic (+) and nonpolar (ϕ) amino acids are conserved residues in the core D-domain motif L/I/V-X-L/I/V. Amino acid positions of the most 5′ residues of the D-domains are indicated with numbers in the left of each amino acid sequence respectively. (B, C) p42 ERK2 *in vitro* kinase assays for cyclin D1 (upper panels). (B) Wild type or T286A mutant recombinant GST- full length Cyc D1 protein was mixed with ^32^P-ATP in the kinase assay reaction buffer in the presence or absence of purified ERK2. Reactions were performed at 30°C for 30 min and stopped by adding sample loading buffer. Samples were separated with SDS-PAGE and ^32^P-uptake was detected by autoradiography. Immunoblotting (IB) analysis using antibodies to cyclin D1 is provided as a reference to show substrate amounts. (C) GST alone (lane 1), GST-C-terminal WT Cyc D1 fusion protein retaining the binding site of MAPK (amino acids 165-295, lane 2), T286A (lane 3) and a complete deletion of the D-domain ΔD, lane 4) were used. IB analysis using antibodies to GSTs provided as a reference to show substrate amounts. (D) Immunoprecipitation (IP) and IB analysis following ectopic expression of Flag-tagged ERK2 together with either HA-tagged WT or ΔD Cyc D1 in HCT 116 colon cancer cells. A Western blot for input controls (10% total lysates) is also shown. (E) Western blot analysis with Thr286 phosphorylated cyclin D1 (pCycD1 Thr286) and total Cyc D1 following transfection of various forms of HA-tagged Cyc D1 expression vectors in HCT 116 colon cancer cells. (F, G) *In vitro* ubiquitination assays using HeLa cell extracts Fraction II as a source of the enzymes necessary to conjugate ubiquitin to substrates and ATP. (F) GST-full length Cyc D1 WT, T286A, or ΔD were used for a reaction either with recombinant ERK2 (lanes 3–5) or without it (lanes 1–2). Samples were separated by SDS-PAGE and immunoblotted with a cyclin D1 antibody. ATP was added to all lanes, and no ubiquitin was added to lane 1. (G) GST-full length Cyc D1 WT was used with or without ubiquitin. After SDS-PAGE separation, immunobloting was performed with antibodies to cyclin D1 (lanes 1, 2) or ubiquitin (lanes 3, 4). (H, I) Pulse-chase analysis of cyclin D1 in HCT 116 cells after exposure to U0126. Exponentially growing HCT 116 cells were treated with DMSO or 10 µM U0126 for 30 min. (H) Cells were pulse-labeled with ^35^S-methionine and were chased for the indicated times. Cyclin D1 was immunoprecipitated and then analyzed with SDS-PAGE. Autoradiography was performed. (I) Levels of metabolically labeled-cyclin D1. (J) Western blot analysis with pCycD1 Thr286, total cyclin D1, phosphorylation specific ERK (pERK), and total ERK antibodies. (K) Cell cycle distributions are shown.

MAPK activity is elevated in all three cancer cell lines examined (data not shown; [ref.4]). We investigated the possibility that the Ras/Raf/MEK/ERK MAPK signaling cascade might regulate the phosphorylation of cyclin D1 residue Thr286, which is followed by proline (Fig. 2A; [refs. 8], [9]).

ERK/MAPK is a proline (Pro)-directed protein kinase [26]. It requires a kinase docking site (or D-domain) on its substrate to increase phosphorylation efficiency (Fig. 2A; [ref. 27]). D-domains have been found in various ERK substrates, such as Elk-1, Sap-1, Sap-2, Ets-1 and c-Myc (Fig. 2A; [refs. 27], [28]). We searched for a D-domain on cyclin D1 with Motif Scan software (http://scansite.mit.edu). Through a series of stringent searches, we identified a highly significant (within 0.041 percentile) D-domain in amino acids 179-193 (Fig. 2A), suggesting that the Ras/Raf/MEK/ERK MAPK signaling cascade might be responsible for cyclin D1 phosphorylation.

To test the possibility that purified ERK/MAPK might phosphorylate recombinant cyclin D1, purified ERK2 was surely used to phosphorylate a glutathione *S*-transferase (GST)-cyclin D1 fusion protein (Figs. 2B and C). Purified ERK2 efficiently phosphorylated GST-full-length wild type (WT) cyclin D1 (Fig. 2B, lane 2). In contrast, ERK2 failed to phosphorylate T286A, a cyclin D1 mutant (Fig. 2B, lane 4), suggesting that Thr286 is the major phosphorylation site of ERK/MAPK. Identical results were obtained in the presence of purified CDK4/6; ERK was able to phosphorylate cyclin D1 at Thr286, not only in the monomeric form but also within cyclin D1-CDK4/6 complexes (data not shown).

To determine if ERK/MAPK requires the D-domain for efficient cyclin D1 phosphorylation at Thr286, we performed *in vitro* kinase assays using a complete deletion of the D-domain (ΔD) from the GST-C-terminal cyclin D1 fusion protein. This fusion protein retains the MAPK binding site. Fig. 2C shows that purified ERK2 effectively phosphorylated WT cyclin D1 (lane 2), but not the T286A and ΔD mutants (lanes 3 and 4).

These results strongly suggest that MAPK interacts with cyclin D1 through its D-domain to phosphorylate Thr286. To confirm this possibility, we performed an immunoprecipitation and immunoblotting (IP-IB) analysis following ectopic expression of Flag-tagged ERK2 with either HA-tagged WT or ΔD cyclin D1 in HCT 116 colon cancer cells (Fig. 2D). ERK2 associated well with WT cyclin D1 (lane 2) and poorly with ΔD cyclin D1 (lane 3). To establish the importance of MAPK in the phosphorylation of cyclin D1 at Thr286 in the HCT 116 cells, we transfected the cells with various forms of cyclin D1 expression vectors (Fig. 2E). Ectopic expression of cyclin D1 was distinguished from endogenous expression by the reduced mobility of HA-tagged cyclin D1. We analyzed the phosphorylation status of exogenous cyclin D1 expression at Thr286. Cyclin D1 phosphorylation was significantly reduced by the deletion of the D-domain (lane 4). These observations suggested that the majority of Thr286 phosphorylation in HCT 116 cells is through MAPK activity.

### Ras/MAPK-mediated ubiquitination and degradation of cyclin D1 is directly linked to the association of MAPK/ERK with cyclin D1

We next investigated whether MAPK-mediated phosphorylation of cyclin D1 may lead ubiquitination of cyclin D1 *in vitro* (Figs. 2F and G). We used a ubiquitination assay system that uses Fraction II HeLa cell extracts as a source of the enzymes necessary to conjugate ubiquitin to substrates and ATP [29]. Polyubiquitination of cyclin D1 (Fig 2F, lanes 1 and 2) was enhanced further by ERK2 (Fig. 2F, lane 3 and Fig. 2G, lanes 2 and 4). Slower migrating bands were not detected in the absence of ubiquitin (Fig. 2G, lanes 1 and 3), suggesting that they consist of polyubiquitinated forms of cyclin D1 (Fig. 2G, lanes 2 and 4). We believe that polyubiquitination required direct interaction of ERK2 with cyclin D1 and the phosphorylation of cyclin D1 at Thr286, because ubiquitination was largely prevented in the D-domain deletion mutant form (ΔD) and the alanine for Thr286 substitution (T286A) of cyclin D1 (Fig. 2F, lanes 4 and 5).

### The stability of cyclin D1 protein is regulated by ERK/MAPK activities in HCT 116 cancer cells

We also determined the contribution of ERK/MAPK to the stability of cyclin D1 in cancer cells. We performed pulse-chase analysis on metabolically labeled-cyclin D1 after inhibiting MAPK activities (Figs. 2H–K; [refs. 30], [31]). Exponentially growing HCT 116 cells were treated with U0126 for 30 min, which significantly depleted the phosphorylated form of ERK (pERK) and cyclin D1 at Thr286 (pCyc D1 Thr286) without affecting the cell cycle profile (Figs. 2J and K). Levels of metabolically labeled-cyclin D1 were estimated by quantitative scanning as described above (Fig. 2I). Reducing MAPK activity increased the half-life of cyclin D1 from 22.5 min to 54.6 min. These data indicate that the stability of cyclin D1 is regulated by ERK/MAPK activity in cancer cells.

### Cyclin D1 stability is regulated through the SCF or the SCF-like pathway

**Figure 3:**
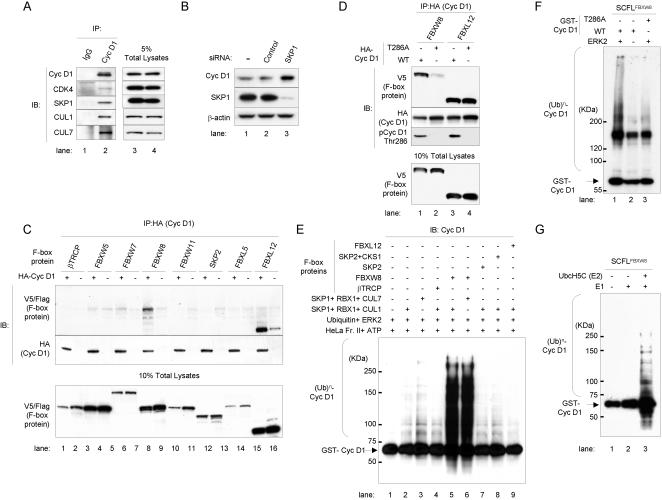
FBXW8 ubiquitinates cyclin D1 in a Thr286 phosphorylation-dependent manner. (A) IP-IB analysis (left). Protein from exponentially growing HCT 116 cells was precipitated with antibodies to cyclin D1 or IgG. Immunoprecipitates were subjected to SDS-PAGE and sequentially blotted with cyclin D1, CDK4, SKP1, CUL1 and CUL7 antibodies. IB analysis with 5% of total cell lysates was provided as a control (right). (B) IB analysis following depletion of SKP1 expression for 48 hrs after treatment with SKP1 siRNA double-strand oligonucleotides in HCT 116 cells. Non-targeting siRNA (Control) and mock transfection (−) served as controls. (C) IP-IB analysis. Twenty-six F-box full-length encoding cDNAs were cloned into V5 or Flag epitope tag expression vectors. These V5 or Flag-tagged F-box protein DNA plasmids were transfected together with HA-tagged cyclin D1 (HA-Cyc D1) and CDK4 expression vectors into T98G cells. Cells were collected 24 hrs later. Samples were precipitated with a HA epitope tag antibody. Immunoprecipitates were subjected to SDS-PAGE and subsequently stained with V5 or Flag (F-box proteins), HA (Cyc D1) antibodies. IB analysis with 10% of total cell lysates was provided (bottom). (D) IP-IB analysis (top). V5-tagged F-box protein DNA plasmids were transiently transfected together with either HA-tagged cyclin D1 (Cyc D1) wild type (WT) or T286A mutant, and CDK4 expression vectors in T98G cells respectively. Samples were precipitated with a HA epitope-tag antibody. Immunoprecipitates were subjected to SDS-PAGE and subsequently blotted with V5 (F-box proteins), HA (Cyc D1), and Thr286 phosphorylated cyclin D1 (pCyc D1 Thr286) antibodies. IB analysis with 10% of total cell lysates was provided for comparison (bottom). (E) *In vitro* ubiquitination assay. *In vitro* translated F-box proteins with recombinant GST-full-length cyclin D1 (Cyc D1) wild type, HeLa cell extracts Fraction II with ATP, Ubiquitin and ERK2, and *in vitro*-translated either SKP1, RBX1 and CUL1, or SKP1, RBX1 and CUL7 proteins were incubated at 30°C for 2 hrs. Samples were separated by SDS-PAGE and immunoblotted with a cyclin D1 antibody. (F) *In vitro* polyubiquitination of cyclin D1 through the SCF-like (SCFL) complex FBXW8 (SKP1-CUL7-FBXW8-RBX1/SCFL^FBXW8^). WT or T286A GST- Cyc D1 was incubated in the presence of purified ERK2 (lanes 1, and 3) or its absence (lane 2) at 30°C for 2 hrs. Samples were separated by SDS-PAGE and immunoblotted with a cyclin D1 antibody. Asterisk indicates non-specific bands. (G) Reconstitution of polyubiquitination of cyclin D1 through SCFL^FBXW8^ *in vitro* using purified E1 and E2. GST-WT Cyc D1 was incubated with recombinant SCFL^FBXW8^ in the presence or absence of E1 and E2/UbcH5C. Samples were separated by SDS-PAGE and immunoblotted with a cyclin D1 antibody.

Because of the strict specificity of E3 ligases and their substrates [18], cyclin D1 is likely to have its own E3 ligase. We hypothesized that cyclin D1 proteolysis is mediated by the SCF E3 ligases or an SCF-like complex of E3 ligases, where an F-box protein determines the specificity for its substrate. To test this idea, we performed IP-IB analysis (Fig. 3A). Cyclin D1 from exponentially growing HCT 116 cells was immunoprecipitated and sequentially blotted with antibodies to cyclin D1, CDK4, SKP1, CUL1 and CUL7. Cyclin D1 associated with SKP1, CUL1 or CUL7, and CDK4, suggesting that its proteolysis is mediated by the SCF (SKP1-CUL1-F-box protein) or the SCF-like (SKP1-CUL7-FBXW8) complex of E3 ligases.

To test whether levels of cyclin D1 are mainly regulated by the SCF or the SCF-like pathway, we performed an immunoblot analysis 48 hrs after depleting SKP1 expression with siRNA double-strand oligonucleotides in HCT 116 cells (Fig. 3B). siRNA for SKP1 significantly reduced SKP1 expression and resulted in accumulation of cyclin D1. These observations strongly support the idea that cyclin D1 stability is regulated through the SCF or the SCF-like pathway.

### FBXW8, an F-box protein, specifically associates with cyclin D1 in a Thr286 phosphorylation dependent manner

We next identified the protein responsible for cyclin D1 stability. We tested candidate human F-box protein genes to identify the unique E3 ubiquitin ligase for cyclin D1. Substrate specificity of SCF complexes occurs through protein-protein interaction domains that are often tryptophan-aspartic acid (WD) 40 motifs or leucine-rich repeats (LRR) within F-box proteins [20], [21]. We searched the NCBI databases for human F-box proteins with WD40 or LRR motifs. We found approximately 70 potential genes. Among these, 9 had WD40 repeat motifs and 17 had LRR motifs.

We obtained these genes by first performing reverse transcriptase-PCR (RT-PCR) using total RNA from HEK 293, HCT 116 or WI-38 cells. The full-length cDNAs we retrieved were cloned into V5 or Flag epitope tag expression vectors. To address whether any of the products of these genes could recognize cyclin D1, we transiently transfected DNA plasmids for the V5 or Flag-tagged F-box proteins into T98G cells with or without N-terminal HA-tagged cyclin D1 and CDK4 expression vectors (Fig. 3C). After 24 hrs, the cells were collected and performed IP-IB analysis. The samples were precipitated with an HA epitope tag antibody and stained with Flag (FBXW7 and FBXL5) or V5 (others), and cyclin D1 antibodies. Panel C shows cyclin D1 associating with two F-box proteins, FBXW8 (lane 7) and FBXL12 (lane 15). FBXW8 possessed WD40 motifs and FBXL12 had LRR motifs.

Because F-box protein substrates must be phosphorylated [19], we tested whether FBXW8 and FBXL12 recognize cyclin D1 in a Thr286 phosphorylation-dependent manner. We transiently transfected T98G cells with V5-tagged F-box protein DNA plasmids and cyclin D1 (wild type or T286A mutant) and CDK4 expression vectors. Samples were precipitated with a HA epitope tag antibody and blotted with V5, HA and Thr286 phosphorylated cyclin D1 antibodies (Fig. 3D). FBXW8 was associated with both cyclin D1 wild type and the T286A mutant, but the majority was bound to wild type, which was mostly phosphorylated at Thr286. In contrast, we did not see a significant difference between wild-type cyclin D1 and the mutant in association with FBXL12. These results suggest that FBXW8 recognizes cyclin D1 in a Thr286 phosphorylation-dependent manner, but FBXL12 does not. Consistent with this finding, FBXL12 was not involved in cyclin D1 polyubiquitination *in vitro* (Fig. 3E, lane 9). We concluded that FBXW8 may play a role in cyclin D1 stability.

### FBXW8 ubiquitinates cyclin D1 in a Thr-286 phosphorylation dependent manner

We investigated whether *in vitro* ubiquitination of cyclin D1 requires FBXW8 (Fig. 3E). We incubated each *in vitro*-translated F-box protein with recombinant GST-cyclin D1 (Cyc D1), fraction II HeLa cell extracts with ATP, ubiquitin and ERK2, and either *in vitro*-translated SKP1, RBX1 and CUL1, or SKP1, RBX1 and CUL7 proteins. Next, we blotted them with a cyclin D1 antibody. Cyclin D1 ubiquitination was detected in the combinations of FBXW8 with SKP1, CUL1, and RBX1 (lane 5), or FBXW8 with SKP1, CUL7, and RBX1 (lane 6). However, polyubiquitinated bands were not increased in other combinations. To confirm that the SCF complexes were assembled properly upon *in vitro* translation, we performed immmunoprecipitation with each F-box protein in the ^35^S-labeled *in vitro* translated samples (not shown) and tested whether the complexes containing β-TRCP were functional for polyubiquitination of β-catenin (Fig. S1 [E]). Our results suggest that cyclin D1 ubiquitination involves FBXW8.

We next investigated whether *in vitro* ubiquitination of cyclin D1 through the SCF-like (SCFL) complex FBXW8 (SKP1-CUL7-FBXW8-RBX1/SCFL^FBXW8^) requires phosphorylation of cyclin D1 at Thr286 (Fig. 3F). Polyubiquitination through SCFL^FBXW8^ was dramatically reduced by the depletion of ERK2 (lane 2). Furthermore, cyclin D1 polyubiquitination was largely prevented by the alanine-for-Thr286 substitution (T286A, lane 3), suggesting that phosphorylation of cyclin D1 at Thr286 is necessary for ubiquitination by SCFL^FBXW8^. These data are in good accordance with our observation that FBXW8 specifically associates with cyclin D1 in a Thr286 phosphorylation-dependent manner.

Finally, we reconstituted cyclin D1 polyubiquitination *in vitro* using purified E1 and E2 enzymes (Fig. 3G). SCFL^FBXW8^ promotes UbcH5C-catalyzed polyubiquitin chain assembly [22]. Consistent with this fact, the V5 immunoprecipitates containing SCFL^FBXW8^ exhibited significant E3 activities for polyubiquitination of cyclin D1 in the presence of both E1 and E2/UbcH5C (lane 3), and no activity in the absence of E1 or both E1 and E2 (lanes 1 and 2). Taken together, these data indicate that 1) cyclin D1 can be ubiquitinated by FBXW8 and 2) that this process is dependent on Thr286 phosphorylation of cyclin D1 by ERK/MAPK.

### Cyclin D1 levels are regulated by FBXW8

**Figure 4:**
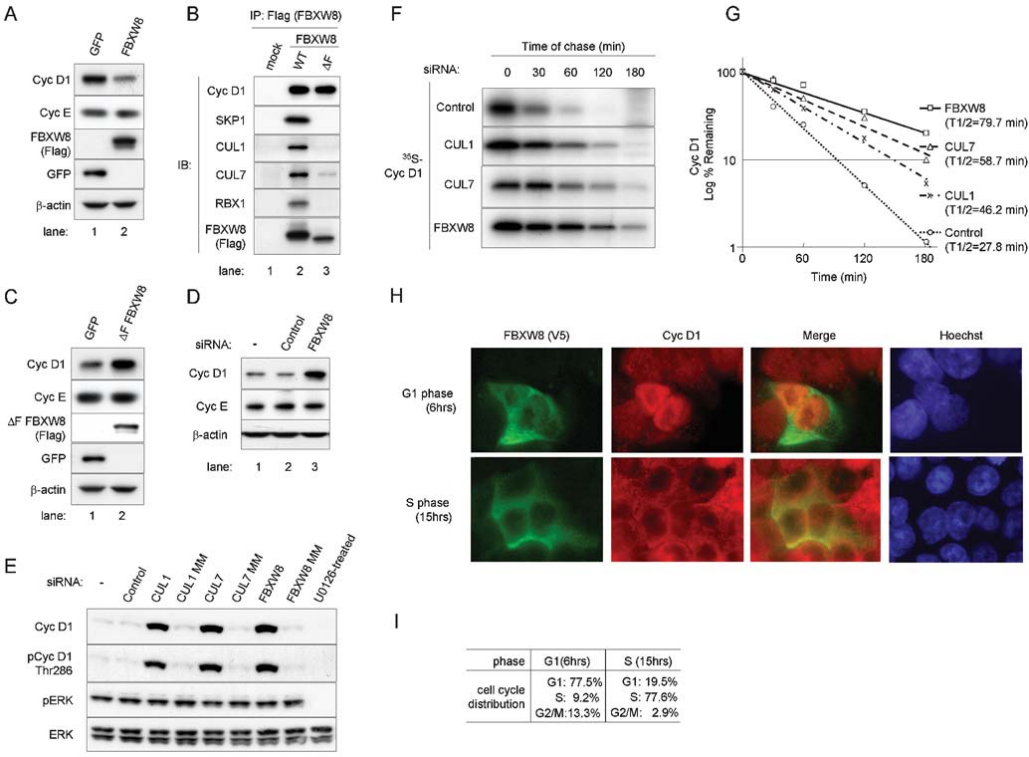
The stability of cyclin D1 is regulated by FBXW8 complexes through the ubiquitin-proteasome pathway. (A, C) IB analysis. HCT 116 cells were infected with a retrovirus expressing FBXW8 (A), a ΔF mutant form (ΔF FBXW8, Panel C) or a control retrovirus expressing GFP (A, C). Forty-eight hours later, cells were harvested and Western blot analysis was performed with antibodies to cyclin D1, cyclin E, Flag (FBXW8 and ΔF FBXW8), GFP and β-actin. (B) IP–IB analysis. Empty (mock) or Flag-tagged WT FBXW8, and ΔF FBXW8 DNA plasmids were transiently transfected in T98G cells. Samples were precipitated with a Flag epitope tag antibody. Immunoprecipitates were subjected to SDS-PAGE and subsequently blotted with antibodies to cyclin D1, SKP1, CUL1, CUL7, RBX1 and Flag (F-box proteins). (D) IB analysis following depletion of FBXW8 expression for 48 hrs through siRNA in HCT 116 cells. Non-targeting siRNA (Control) and mock transfection (−) were controls. (E–G) Knockdown of FBXW8 or its partner CUL1 or CUL7 through siRNA stabilizes cyclin D1 expression in HCT 116 cells. (E) IB analysis following depletion of CUL1, CUL7 or FBXW8 expression for 48 hrs through siRNA or mismatch (MM) oligonucleotides in HCT 116 cells. Non-targeting siRNA (Control) and mock transfection (−) were controls. (F, G) Pulse-chase analysis of cyclin D1 following depletion of CUL1, CUL7 or FBXW8 expression for 48 hrs through siRNA in HCT 116 cells. Control cells were treated with non-targeting siRNA. Cells were pulse-labeled with ^35^S-methionine for an hour, chased with cold methionine as indicated, and then lysed. Cyclin D1 was immunoprecipitated and analyzed via SDS-PAGE. (G) Levels of metabolically labeled-cyclin D1 were estimated by quantitative scanning using Quantity One software and plotted graphically to determine the half-life of cyclin D1. (H, I) Immunofluorescence. Nuclei were visualized with Hoechst dye. Subcellular localization of E3 ligase. HCT 116 cells were transfected with V5 epitope-tagged FBXW8 pcDNA3. Cells were synchronized 24 hrs later by sequential manipulation of serum starvation and stimulation. At 6 hrs (G1 phase) or 15 hrs (S phase), cells were fixed. Immunofluorescence was performed with a V5 epitope tag antibody followed by Alexa Fluor 488-conjugated anti-mouse IgG antibody (green) and a rabbit cyclin D1 polyclonal antibody followed by Alexa Fluor 594-conjugated anti-rabbit IgG antibody (red). (I) Cell cycle distributions.

We tested whether ectopic expression of FBXW8 reduces levels of endogenous cyclin D1 in cultured cells. We infected HCT 116 cells with retroviruses expressing FBXW8, FBXW7, or GFP as a control (Fig. 4A and Fig. S1 [F]).

As Fig. 4A shows, overexpression of FBXW8 reduced endogenous expression of cyclin D1, but did not significantly change expression profiles of cyclin E. In contrast, FBXW7 inhibited expression of cyclin E, but not cyclin D1 (Fig. S1 [F]; [ref. 32]). Similar profiles were obtained from SW480, U-2 OS, and T98G cells (not shown).

We also investigated whether overexpression of a dominant-negative form of FBXW8 could cause cyclin D1 accumulation in exponentially growing cells. The F-box deletion ΔF) mutant form of FBXW8 is considered to be a dominant-negative because it can bind to cyclin D1 but barely associates with SKP1, CUL1 and CUL7 (Fig. 4B, lane 3), and therefore does not bring cyclin D1 into the ubiquitin-proteasome pathway. We infected HCT 116 cells with retroviruses expressing ΔF FBXW8 or GFP (Fig. 4C). There was significant cyclin D1 accumulation following ΔF FBXW8 expression. In contrast, an ectopically expressed dominant-negative form of FBXW8 did not significantly change levels of cyclin E (Fig 4C). Similar observations were obtained from SW480 and T98G cells (not shown).

We confirmed this finding by determining whether siRNA-induced depletion of endogenous FBXW8 expression could cause cyclin D1 to accumulate in HCT 116 cells (Fig. 4D). We treated cycling HCT 116 cells with control or FBXW8 siRNA for 48 hrs. FBXW8 inhibition was verified RT-PCR analysis. We observed approximately 95% inhibition of FBXW8 in comparison to the control sample (see Fig. S1 [G]). We observed significant cyclin D1 accumulation in the sample treated with FBXW8 siRNA (Fig. 4D, lane 3) and no effect on cyclin E levels. We concluded that cyclin D1 levels are regulated by FBXW8 in the cancer cells tested here.

### The stability of cyclin D1 is regulated through complexes containing FBXW8

FBXW8 associates with CUL1 or CUL7 and forms a complex with SKP1 and RBX1 (Fig. 4B lane 2; [ref. 22], [23]), suggesting that CUL1 and CUL7 define the stability of cyclin D1 through FBXW8. Given that depleting FBXW8 from cultured cells increased cyclin D1 levels, reducing CUL1 or CUL7 should give the same result. We treated HCT 116 cells with siRNA for 48 hrs to knock down expression of CUL1 or CUL7 (Fig. 4E). In parallel, we used RT-PCR to confirm that the siRNA transfection was working efficiently (Fig. S1 [G]). The siRNAs for CUL1, CUL7, or FBXW8 reduced expression of their respective genes, resulting in accumulation of cyclin D1, which was mostly phosphorylated at Thr286 (Fig. 4E). The effect was achieved without affecting MAPK activities (pERK) in the first 48 hrs of siRNA treatment (Fig. 4E). Comparable data were obtained from SW480, U-2 OS, and T98 cells (not shown).

To confirm that cyclin D1 accumulation was due to increased cyclin D1 stability, we performed pulse-chase analysis on metabolically labeled-cyclin D1 after using siRNA to deprive HCT 116 cells of FBXW8, CUL1, or CUL7 (Fig. 4F). Levels of metabolically labeled-cyclin D1 were estimated as described (Fig. 4G). Reducing FBXW8, CUL7 or CUL1 led to a stabilization of cyclin D1. The half-life of cyclin D1 was 27.8 min in control cell, and was extended by FBXW8, CUL7, or CUL1 siRNA treatment (T_1/2_ = 79.7, 58.7, or 46.2 min respectively). These data confirmed that accumulation of cyclin D1 through depletion of FBXW8, CUL1, or CUL7 was caused by increased cyclin D1 stability. We conclude that cyclin D1 stability is regulated by complexes containing FBXW8, through the ubiquitin-proteasome pathway.

### Increased cyclin D1 degradation in cancer cells is linked to increased cyclin D1-E3 ligase association

We demonstrate that cyclin D1 undergoes increased degradation in the cytoplasm during S phase in a variety of cancer cells. We hypothesized that enhanced cyclin D1 degradation in S phase is associated with an increase in the association of E3 ligase with cyclin D1. To test this hypothesis, we determined the subcellular localizations of FBXW8 and cyclin D1 during the cell cycle in cancer cells (Figs. 4H and I). HCT 116 cells were transfected with V5 epitope-tagged FBXW8. The cells were rendered quiescent 24 hours later by serum starvation for a period lasting a further 24 hours, and then stimulated by adding medium containing serum. This process allowed synchronization of cell cycle progression. Cell cycle profiles were determined by flow cytometry (Fig. 4I). At times corresponding to the G1 and S phases, cells were fixed and labeled with fluorescent V5 epitope tags and cyclin D1 antibodies (Fig. 4H). The majority of FBXW8 was expressed in the cytoplasm during G1 and S phase. In contrast, cyclin D1 accumulated in the nucleus during G1 phase and exited into the cytoplasm in S phase, in agreement with previous reports [8], [9], [25]. The separation of FBXW8 and cyclin D1 during G1 phase suggested that ubiquitination and degradation of cyclin D1 is prevented in G1. Conversely, their colocalization during S phase demonstrated that cyclin D1 proteolysis could be increased in the cytoplasm as cells proceed into S phase (Fig. 1G).

### FBXW8-mediated cyclin D1 degradation in the cytoplasm is required for cancer cell proliferation

**Figure 5:**
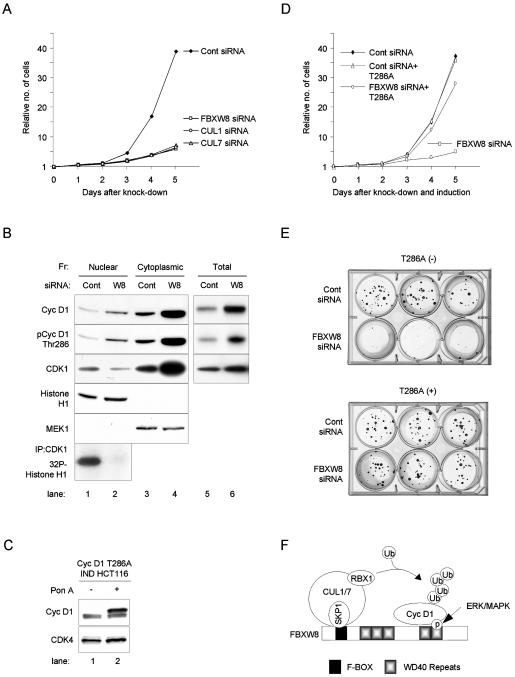
Cyclin D1 degradation in the cytoplasm is essential for cell proliferation. (A) Viable HCT 116 cells after siRNA-mediated knockdown of FBXW8, CUL1, or CUL7 expression. siRNAs were transfected on days 0, 1, 2, and 4. Cells were collected as indicated, stained with trypan blue, and counted with a haemocytometer. (B) Western blot analysis with total and fractionated nuclear proteins. Protein blotting was performed with total cyclin D1 (Cyc D1), pCyc D1 Thr286, CDK1, Histone H1, and MEK1 antibodies. HCT 116 cells were treated with control siRNA (Cont) or FBXW8 siRNA (W8) for 48 hrs. Samples were then fractionated into nuclear or cytoplasmic proteins, or prepared as total cell lysates. The bottom panel shows a CDK1-associated Histone H1 *in vitro* kinase assay using nuclear protein. (C) Generation of cyclin D1 ecdysone-inducible (IND) system in HCT 116 cells. Ectopic expression of HA-tagged T286A was induced in by 10 µM Ponasterone A (Pon A). (D) Viable cells after T286A cyclin D1 induction. T286A IND HCT 116 cells were cultured in the presence of Pon A (T286A) or its absence, and control (Cont) or FBXW8 siRNA. (E) Colony formation assay. One hundred single T286A IND HCT 116 cells were cultured in the presence of Pon A (+) or its absence (−), and control siRNA (Cont) or FBXW8 siRNA. Cells were cultured for 2 weeks, and stained with 0.5% crystal violet containing 20% ethanol. (F) A model of cyclin D1 ubiquitination mediated by the complex containing FBXW8. FBXW8 recognizes cyclin D1 through a WD40 repeat motif in an ERK/MAPK-mediated Thr286 phosphorylation-dependent manner. SKP1 interacts with FBXW8 together with CUL1 or CUL7 via a domain in the N-terminus of FBXW8, called F-box. CUL1 or CUL7 recruits RBX1, which in turn conscripts E2, a ubiquitin-conjugating enzyme, to add a multiubiquitin chain to cyclin D1.

A recent report suggested that cyclin D1 degradation is necessary for efficient DNA synthesis in NIH 3T3 cells [33]. We examined the biological significance of enhanced cyclin D1 degradation in the cytoplasm during S phase in HCT 116 cells. We inhibited cyclin D1 proteolysis in the cytoplasm by using siRNA to knock down E3 ligase components such as FBXW8, CUL1, or CUL7 and counted cells for five days (Fig. 5A). siRNA for FBXW8, CUL1, or CUL7 significantly reduced cell numbers. These data indicate that rapid turnover of cyclin D1 is required for HCT 116 proliferation.

A recent study demonstrated that cytoplasmically expressed cyclin D1 competes with nuclear cyclin D1 and translocates CDK4 from the nucleus to the cytoplasm [34]. The consequence is a growth arrest. We tested whether reduced cell proliferation due to knocking down FBXW8 expression is caused by cyclin D1 accumulation and subsequent cytoplasm sequestration of CDK1 (Fig. 5B). We treated HCT 116 cells with control (Cont) or FBXW8 (W8) siRNA for 48 hrs. Inhibition of FBXW8 expression was verified by a RT-PCR. We subsequently fractionated nuclear and cytoplasmic proteins. Fig. 5B shows that depleting FBXW8 caused cytoplasmic cyclin D1 accumulation. Cyclin D1 was mostly phosphorylated at Thr286, suggesting that cyclin D1 degradation is linked to enhanced phosphorylation of cyclin D1 by MAPK (see Figs. 2H–K). This process resulted in relocalization of CDK1 from the nucleus to the cytoplasm. This caused a dramatic reduction of the nuclear CDK1 kinase activities, as assessed by a CDK1-associated Histone H1 *in vitro* kinase assay (Fig. 5B bottom). These observations indicated that inhibiting rapid turnover of cyclin D1 induced growth arrest.

We examined whether constitutive expression of the nuclear protein cyclin D1 T286A could abrogate the block to cell proliferation caused by siRNA against FBXW8 (Figs. 5C–E). We tested this cyclin D1 mutant because it is resistant to polyubiquitination, and also prevents the nuclear export of cyclin D1 during S phase resulting in constitutive nuclear localization [9]. Importantly, this mutant is functional: ectopically expressed T286A assembled with CDK4 in cultured cells and showed similar levels of kinase activities to wild type cyclin D1 (data not shown; [ref. 7]).

We generated a cyclin D1 ecdysone-inducible (IND) system in HCT 116 cells. Ponasterone A (Pon A) induced ectopic expression of HA-tagged T286A in physiological levels (Fig. 5C). We subsequently counted viable cell numbers for five days (Fig. 5D) and performed a colony formation assay for 2 weeks (Fig. 5E) in the presence (+) or absence (−) of Pon A, and control (Cont) or FBXW8 siRNA. Cell colonies were stained with crystal violet. Figs. 5D and E show that ectopically expressed physiological levels of nuclear protein cyclin D1 T286A dramatically rescued cells from growth arrest. Thus, FBXW8-mediated cyclin D1 degradation is essential for proliferation of HCT 116 cells.

## Discussion

This study has shown that the Ras/Raf/MEK/ERK MAPK signaling cascade promotes cyclin D1 phosphorylation at Thr286, resulting in ubiquitination and degradation of cyclin D1 (Fig. 5F). ERK/MAPK is a proline (Pro)-directed serine or threonine (Ser/Thr) protein kinase [26]. Many proteins contain Ser/Thr-Pro sequences; however, most of them are not phosphorylated by ERK, suggesting that there is a strict relationship in ERK substrate specificity [28]. MAPK requires D-domains on the substrate to increase the efficiency of phosphorylation [27]. In this report, we identified a stringent D-domain at amino acids 179-193 of cyclin D1. Deleting this domain significantly reduced phosphorylation of cyclin D1 in cultured cells and dramatically inhibited ubiquitination of cyclin D1 *in vitro* (Figs. 2E and F), again demonstrating that ERK/MAPK plays a significant role in cyclin D1 regulation, as GSK3β, another proline-directed protein kinase responsible for cyclin D1 phosphorylation at Thr286, should not require the MAPK D-domain for phosphohrylation of its substrates. This possibility suggests that high levels of MAPK activity in cancer cells may make the requirement of GSK3β redundant for cyclin D1 phosphorylation at Thr286. We also found that similar D-domain sequences were conserved in cyclin D1s from mouse, rat, chicken, xenopus, and zebrafish. Alignment of cyclin D1 with other D-type cyclins has shown that Thr286 in cyclin D1 corresponds to Thr280 in cyclin D2 and Thr283 in cyclin D3 in their proximity to the carboxyl termini immediately followed by prolines [8], [9]. In addition, we found very similar sequences of D-domains in the equivalent sites of cyclin D2 and cyclin D3. We therefore suggest that all D-type cyclins can be classified as ERK/MAPK substrates. In fact, cyclin D3 protein shows a very similar expression profile to cyclin D1 during cell cycle progression in colon cancer cells released from quiescence (Figs. S1 [H] and [I]).

We have also demonstrated that ERK/MAPK-mediated cyclin D1 degradation through FBXW8 is required for proliferation of cancer cells (Fig. 5F). Our discoveries therefore suggest that the Ras/MAPK pathway may play an important role in neoplastic transformation through two distinct effects on cyclin D1 expression: the pathway turns on transcription in response to oncogenic signals via the MAPK signaling cascade, leading to accumulation of cyclin D1 and its assembly with CDK4/6. Progression into S phase requires removing cyclin D1 through relocalization and degradation. This process is initiated by sustained MAPK signaling, a feature unique to cancer cells. Thus, our results provide a better understanding of the roles of MAPK and cyclin D1 in cell cycle regulation.

## Materials and Methods

### Cell culture, vectors, and site-directed mutagenesis

Cell lines were obtained from the American Type Culture Collection. CMV-HA tagged ubiquitin, CUL1, CKS1, and Flag tagged CUL7 DNA expression vectors were gifts from Drs. M. Pagano and Z. Q. Pan. pcDNA3 cyclin D1 T286A and cyclin D1 ΔD mutants, as well as F-box deletion (ΔF) mutant forms of FBXW8 and SKP2 were generated by using site-directed mutagenesis (QuickChange and ExSite, Stratagene).

### Small interfering (si) RNAs

The following siRNA oligonucleotides were selected to knock down endogenous expression of FBXW8, SKP1, CUL1, and CUL7: FBXW8 (AAGAUGUGCACAGGUGAGCAA), CUL1 (AAUAGACAUUGGGUUCGCCGU), and CUL7 (AAGGAUGAGAUCUAUGCCAAC). Mismatch oligonucleotides for FBXW8, CUL1, and CUL7 are 8 bp nucleotides different from their target sequences respectively. The siRNAs for SKP1 were from Dharmacon's SMART pool. siRNAs were transfected using Oligofectamine or Lipofectamine (Gibco, Invitrogen). Relative gene expression following siRNA treatment was measured by RT-PCR (UCSF Cancer Center Genome Core Facility) using TaqMan assays (Applied Biosystems).

### Immunoblotting, and immunoprecipitation and immunoblotting (IP-IB) analyses

Total protein preparation, SDS-PAGE, immunoprecipitation and immunoblotting analysis and enhanced chemiluminescence were carried out as described [3], [4]. NE-PER nuclear and cytoplasmic extraction reagents (Pierce) were used for fractionation. The following monoclonal and polyclonal primary and secondary antibodies were used: cyclin D1 (A-12, M-20, Santa Cruz), cyclin D3 (Transduction Laboratory), cyclin E (Ab-1, Calbiochem), ERK1/2 (Promega), phospho-ERK1/2 (E-4, Santa Cruz), CDK1 (Transdcution Laboratory), CDK4 (Transduction Laboratory, or H-303, Santa Cruz), SKP1 (55893, PharMingen), CUL1 (ZL18, Zymed), CUL7 (BL653, Bethyl Laboratories), RBX1 (Ab-1, NeoMarkers), Ubiquitin (P4D1, Santa Cruz), GST (B-14, Santa Cruz), GFP (FL, Santa Cruz), MEK1 (Transduction Laboratory), Histone H1 (AE-4, Santa Cruz), β-actin (Sigma), HA (12CA5, Roche), Flag (M2, Sigma), V5 (Invitrogen), Sheep anti-mouse IgG HRP and Donkey anti-rabbit IgG HRP (Amersham).

### Generation of a cyclin D1 phosphorylation specific antibody

Phospho-specific antibody against Thr286 of cyclin D1 was raised using KLH-conjugated phospho-peptide KDLAC-pT-PTDVR as an antigen in collaboration with Zymed Inc. Rabbits were immunized three times with the peptides and serum was collected after 3 months, and subjected to affinity-purification using affinity gel coupled with phosphorylated peptide. Anti-nonphosphorylated cyclin D1 antibodies were eliminated by affinity-absorption using gel coupled with unphosphorylated peptide (Zymed).

### Immunoprecipitation and immunoblotting analysis

The following antibodies were used for immunoprecipitation; cyclin D1 (A-12 Agarose-conjugated, Santa Cruz), CDK1 (C-19, Santa Cruz), Flag (M2 Agarose-conjugated, Sigma), and HA (Y-11 Agarose-conjugated, Santa Cruz).

### *In vitro* protein kinase assay

Glutathione *S*-transferase (GST) fusion proteins GST-cyclin D1, GST-cyclin D1-CDK4/6 (Cell Signaling) or Histone H1 (Santa Cruz) were used for *in vitro* kinase assays. Reactions were performed at 30°C for 30 min with kinase buffer (50 mM Tris-HCl [pH 8.0]) and 1 mM DTT containing 30 mM ATP and 10 µCi γ-^32^P ATP in the presence of 10 ng recombinant MEK1 activated GST-ERK2 (14-550, Upstate) or CDK1 immune-complexes from cultured cells. Reactions were stopped with sample loading buffer. Samples were separated by SDS-PAGE and ^32^P uptake was detected by autoradiography.

### Reconstitution of cyclin D1 polyubiquitination *in vitro*

Recombinant SCFL^FBXW8^ were prepared from transfected HEK293 cells. Equal amounts of SCFL^FBXW8^ immune-complexes were mixed with 1 µg GST-full-length Cyc D1 WT protein in the presence of 30 ng recombinant active ERK2 (Upstate) and 0.5 mM ATP for 30 min on ice to allow binding. To the mixture was added 50 ng E1, 100 ng E2 (UbcH5c), 2 µg ubiquitin, and 1 µg ubiquitin aldehyde (all from BostonBiochem). Reactions were performed with a buffer containing 50 mM Tris-HCl (pH 8.0), 1 mM DTT, 5 mM MgCl_2_, 0.5 mM EDTA, 1.5 mM ATP in the presence of 10% glycerol at 30°C for 1 hour and terminated by boiling for 5 min with SDS sample loading buffer. Samples were separated by SDS-PAGE and immunoblotted with cyclin D1 antibody (A-12, Santa Cruz).

## Supporting Information

Figure S1 (A, B) Expression profile of cyclin D1 during cell cycle progression after release from quiescence. WI-38 cells (A) and T98G cells (B). (C) Western blot analysis using nuclear (N) and cytoplasmic (C) fraction (Fr.) proteins extracted from cell lysates collected in Figure 1F, lane 3. The membrane was stained with histone H1, MEK1, and cyclin D1. (D) Immunoprecipitation-immunoblot analysis. HCT 116 cells were transfected with ubiquitin cDNA and synchronized to S phase through sequential manipulation of serum starvation and stimulation. Cells were treated with Leptomycin B (LMB) for 3 hours to inhibit nuclear-to-cytoplasmic localization of cyclin D1 and treated with MG132 for 1 hour before harvesting. Nuclear protein (N) was fractionated and immunoprecipitated with a cyclin D1 antibody and immunoblotted with a HA antibody (upper panel) or a cyclin D1 antibody (lower panel). Asterisk: background non-specific bands. (E) In vitro ubiquitination assay. *In vitro* translated F-box proteins with recombinant GST-β-catenin (Upstate), HeLa cell extracts Fraction II with ATP, Ubiquitin and GSK3β, and *in vitro*-translated SKP1, RBX1 and CUL1 were incubated at 30°C for 2 hours. Samples were separated by SDS-PAGE and immunoblotted with a β-catenin antibody. (F) Immunoblot analysis. HCT 116 cells were infected with a retrovirus expressing FBXW7/CDC4 or a control retrovirus expressing GFP. Cells were harvested 48 hrs and Western blot analysis was performed with antibodies to cyclin D1, cyclin E, Flag (FBXW7), GFP and CDK4. (G) Summary of RT-PCR following depletion of CUL1, CUL7 or FBXW8 expression for 48 hrs through siRNA or mismatch (MM) oligonucleotides in HCT 116 cells (see Fig. 4E). Non-targeting siRNA was provided as control. Relative gene expression is shown. (H, I) Expression profile of cyclin D3 protein during cell cycle progression after release from quiescence. WI-38 cells (H) and HCT 116 cells (I).
(2.44 MB TIF)
