# Supplementary figures and images for: A Critical Role for FBXW8 and MAPK in Cyclin D1 Degradation and Cancer Cell Proliferation
Source: PLoS One. 2006 Dec 27;1(1):e128. doi: 10.1371/journal.pone.0000128 (PMC1762433; doi:10.1371/journal.pone.0000128)

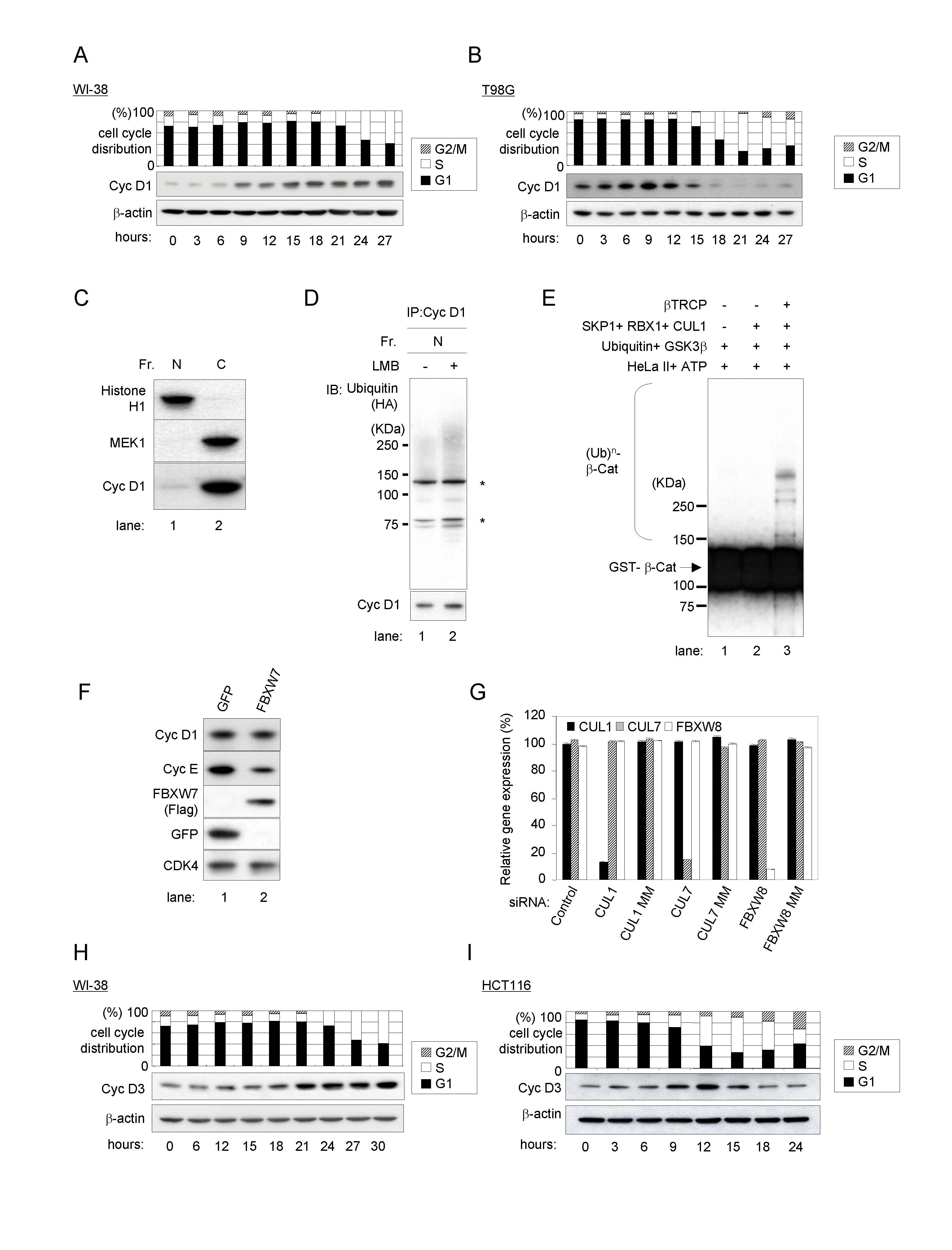

Supplement: Figure S1 — (A, B) Expression profile of cyclin D1 during cell cycle progression after release from quiescence. WI-38 cells (A) and T98G cells (B). (C) Western blot analysis using nuclear (N) and cytoplasmic (C) fraction (Fr.) proteins extracted from cell lysates collected in Figure 1F, lane 3. The membrane was stained with histone H1, MEK1, and cyclin D1. (D) Immunoprecipitation-immunoblot analysis. HCT 116 cells were transfected with ubiquitin cDNA and synchronized to S phase through sequential manipulation of serum starvation and stimulation. Cells were treated with Leptomycin B (LMB) for 3 hours to inhibit nuclear-to-cytoplasmic localization of cyclin D1 and treated with MG132 for 1 hour before harvesting. Nuclear protein (N) was fractionated and immunoprecipitated with a cyclin D1 antibody and immunoblotted with a HA antibody (upper panel) or a cyclin D1 antibody (lower panel). Asterisk: background non-specific bands. (E) In vitro ubiquitination assay. In vitro translated F-box proteins with recombinant GST-β-catenin (Upstate), HeLa cell extracts Fraction II with ATP, Ubiquitin and GSK3β, and in vitro-translated SKP1, RBX1 and CUL1 were incubated at 30°C for 2 hours. Samples were separated by SDS-PAGE and immunoblotted with a β-catenin antibody. (F) Immunoblot analysis. HCT 116 cells were infected with a retrovirus expressing FBXW7/CDC4 or a control retrovirus expressing GFP. Cells were harvested 48 hrs and Western blot analysis was performed with antibodies to cyclin D1, cyclin E, Flag (FBXW7), GFP and CDK4. (G) Summary of RT-PCR following depletion of CUL1, CUL7 or FBXW8 expression for 48 hrs through siRNA or mismatch (MM) oligonucleotides in HCT 116 cells (see Fig. 4E). Non-targeting siRNA was provided as control. Relative gene expression is shown. (H, I) Expression profile of cyclin D3 protein during cell cycle progression after release from quiescence. WI-38 cells (H) and HCT 116 cells (I). (2.44 MB TIF) [file pone.0000128.s001.tif]
